# Association of blood pressure with cognitive function at midlife: a Mendelian randomization study

**DOI:** 10.1186/s12920-020-00769-y

**Published:** 2020-08-26

**Authors:** Daokun Sun, Emy A. Thomas, Lenore J. Launer, Stephen Sidney, Kristine Yaffe, Myriam Fornage

**Affiliations:** 1 Brown Foundation Institute of Molecular Medicine, McGovern Medical School, The University of Texas Health Science Center at Houston, 1825 Pressler Street, Houston, TX 77030 USA; 2 Laboratory of Epidemiology and Population Science, National Institute on Aging, National Institutes of Health, Bethesda, MD USA; 3 Kaiser Permanente Northern California Division of Research, Oakland, CA USA; 4 Departments of Psychiatry and Neurology, University of California San Francisco School of Medicine, San Francisco, CA USA; 5 Human Genetics Center, School of Public Health, The University of Texas Health Science Center at Houston, Houston, TX USA

**Keywords:** Mendelian randomization, Blood pressure, Cognitive disorders, Risk factors, Dementia

## Abstract

**Background:**

Whether high blood pressure has a causal effect on cognitive function as early as middle age is unclear. We investigated whether high blood pressure (BP) causally impairs cognitive function at midlife using Mendelian Randomization (MR).

**Methods:**

We applied a two-sample MR approach to investigate the causal relationship between BP and midlife cognitive performance measured by the Digit Symbol Substitution Test (DSST), Rey Auditory Verbal Learning Test (RAVLT), and Stroop Interference test. We used a total of 109 genetic polymorphisms with established associations with BP as instrumental variables and estimated gene-cognitive function association in 1369 middle-aged adults (Mean age (SD): 50.8 (3.3), 54.0% women) from the CARDIA study.

**Results:**

A 10 mmHg increment in genetically-predicted systolic, diastolic, or pulse pressure was associated with a 4.9 to 7.7-point lower DSST score (*P* = 0.002, SBP; *P* = 0.005, DBP and *P* = 0.008, PP), while a 10 mmHg increment in genetically-predicted SBP was associated with a 0.7 point lower RAVLT and a 2.3 point higher Stroop (*P* = 0.046 and 0.011, respectively).

**Conclusions:**

This MR analysis shows that high BP, especially SBP, is causally associated with poorer processing speed, verbal memory, and executive function during midlife. These findings emphasize the need for further investigation of the role and mechanisms of BP dysregulation on cognitive health in middle age and perhaps, more broadly, across the lifespan.

## Background

Hypertension is one of the long-established modifiable risk factors for age-related dementia [1]. Several studies have reported that high blood pressure (BP) developed by middle age is associated with cognitive decline [2, 3] and with global and domain-specific cognitive impairment in late life [4, 5]. However, other studies have reported non-monotonic or null relationships between high BP and late-life cognitive function [6, 7]. The inconsistency of results may be due to differences in study populations, study design, and methodological aspects. Reverse causation may also play a role. Randomized clinical trials (RCTs) that examine the long-term effects of elevated blood pressure on cognitive ability would represent a gold standard but are costly and difficult to conduct since only randomization based on anti-hypertensive treatment rather than on BP level can be achieved. Early clinical trials designed to evaluate the cognitive benefits of antihypertensive treatment showed conflicting results, most of which were not in favor of beneficial effects [8]. The more recent SPRINT MIND trial reported that, in older adults at increased risk of cardiovascular disease, intensive systolic BP control reduced the risk of mild cognitive impairment (MCI) as well as the combined occurrence of MCI or dementia [9]. Whether the possible benefits of BP control in older adults extend earlier in life is unknown.

We employed a two-sample MR approach to expand existing knowledge about the effect of high BP burden on cognitive health during middle age [10], a pivotal period in the life course when cognitive function begins to decline among healthy adults [11].

## Methods

### Study population

Participants were from the Coronary Artery Risk Development in Young Adults (CARDIA) study, a prospective multi-center cohort study investigating the natural history and etiology of cardiovascular disease in 2637 African-Americans and 2478 European-Americans aged 18–30 years at the time of initial examination in 1985–1986 [12]. Participants were recruited from the community in Birmingham, AL; from selected census tracts in Chicago, IL and Minneapolis, MN; and from the Kaiser-Permanente health plan membership in Oakland, CA. The sample was approximately balanced with respect to race, age, sex, and education groups. Participants have completed nine sequential examinations every 2 to 5 years for 30 years. For this study, we used 1369 subjects of European ancestry who had genome-wide genotype data and phenotypic data for three cognitive tests at Year 25 examination.

### Cognitive assessment

Three standardized tests in CARDIA were administered at Year 25 examination (2010–2011) to assess major aspects of cognitive function by trained and certified technicians following an established protocol as described elsewhere [13] and in the supplemental materials (Additional File 1). Briefly, the battery of tests included the Rey Auditory Verbal Learning Test (RAVLT) (score range: 0–15), a measure of verbal learning and memory [14]; the Digit Symbol Substitution Test (DSST) (Part D) (score range: 0–133), a measure primarily of psychomotor speed; and the Stroop Interference Test (score range: 1–160), a measure of executive function [15]. A higher score in DSST and RAVLT indicates better cognition in the corresponding domain whereas that in the Stroop Interference test reflects poor cognitive performance.

### Instrumental variables and association analysis

A total of 152 single nucleotide polymorphisms (SNPs) previously identified in one of the largest GWAS of BP in individuals of European descent [16] were selected for analysis. These SNPS were selected based on their genome-wide significant association with blood pressure traits (*P* < 5 × 10^− 8^) and evidence of independent replication in multiple studies. From the summary statistics, we obtained the association estimate of each SNP with systolic blood pressure (SBP), diastolic blood pressure (DBP), and pulse pressure (PP) respectively, which had been replicated among 140,886 European participants. Of these, 145 were available in CARDIA. To minimize pleiotropic effects [17], we removed 8 SNPs which overlapped or were in high linkage disequilibrium (LD) (r^2^ > 0.8) with SNPs influencing either known BP risk factors (body mass index (BMI), smoking, and type 2 diabetes), which can serve as confounders; or education, which may bias the gene-cognition association [18]. We also pruned SNPs that were in LD with one another at r^2^ greater than 0.001 within a 10 kb distance. Thus, we used 109 SNPs independently and exclusively associated with BP at genome-wide significance level as instrumental variables (IVs) (99 for SBP, 98 for DBP, and 98 for PP, Table S1). We estimated the allelic effect of each SNP on measures of cognitive performance in 1369 CARDIA participants adjusting for age, sex, and study site. We aligned the direction of SNP-cognitive function association with that in the BP GWAS.

### Statistical analysis

We implemented a multivariable two-sample Mendelian Randomization (MR) approach using the *TwoSampleMR* R package [19] to explore the causal association of genetically-predicted BPs with DSST, RAVLT, and Stroop test’s performance. A summary of our analytical approach is shown in Figure S1 (Additional file 1). As primary analysis, we employed a standard inverse-variance weighted (IVW) method to derive the causal estimates for each variant, which were combined by random-effects meta-analysis. We carried out detection of potentially invalid or influential instruments using radial plots in *RadialMR* [20]*.* We then repeated IVW MR analyses after removing the outliers. We applied additional MR methods including the weighted median method [21], MR-Egger regression [22], and the contamination mixture MR [23] as sensitivity analyses for robust inference.

To examine the effect of potential unbalanced pleiotropy on causal estimates, we employed the MR-Egger intercept test [24], the Mendelian Randomization Pleiotropy RESidual Sum and Outlier (MRPRESSO) test [25] and constructed funnel plots [26]. We evaluated the assumption of “NO Measurement Error” (NOME) by Bowden *I*^*2*^ statistics. We further estimated the heterogeneity of selected IVs by Cochran’s Q test [27]. To preclude the possibility that the causal inference was driven by any individual variant, we performed a leave-one-out analysis for the IVW and MR-Egger methods.

Because of the overlap in genetic instruments for the 3 BP measures (Table S1, Additional file 1) and correlations among cognitive tests, we did not adjust for multiple testing. A *P*-value of association less than 0.05 is considered significant.

## Results

The CARDIA participants were in their fifties (Mean [range] age: 51 [42–57] years) at Year 25. They were well educated but were slightly overweight. One-quarter of them were hypertensive, but less than one fifth were taking anti-hypertension medication. The prevalence of type 2 diabetes and current smoking were low (Table 1).

**Table 1:** Characteristics of 1369 CARDIA participants with cognitive function at Year 25

Variable	Value
Sample size, N	1369
Age (SD) (year)	50.8 (3.3)
Female (%)	739 (54.0)
Center (%)	
Birmingham, AL	273 (19.9)
Chicago, IL	320 (23.4)
Minneapolis, MN	443 (32.4)
Oakland, CA	333 (24.3)
At least college education (%)	917 (67.0)
BMI (SD) (kg/m^2^)	28.3 (6.1)
Type2 Diabetes (%)	124 (9.1)
Current Smoking (%)	165 (12.1)
Systolic Blood Pressure (SD) (mmHg)	114.4 (13.7)
Diastolic Blood Pressure (SD) (mmHg)	70.8 (10.1)
Pulse Pressure (SD) (mmHg)	43.6 (7.6)
Hypertension (%)	341 (24.9)
HBP Rx use (%)	228 (16.7)
DSST (Mean (SD): Min-Max)	75.0 (14.9): 21–125
RAVLT (Mean (SD): Min-Max)	9.4 (3.1): 0–15
Stroop (Mean (SD): Min-Max)	19.3 (8.0): 4–71

*Abbreviations*: CARDIA, the Coronary Artery Risk Development in Young Adults Study; *SD* Standard deviation, *BMI* Body mass index (calculated as weight in kilograms divided by height in meters squared), *HBP Rx* Anti-hypertensive medication, *DSST* Digit Symbol Substitution Test, *RAVLT* Rey Auditory Verbal Learning Test, *Stroop* The Stroop Interference test

The most notable association by the standard IVW method was found between midlife genetically-predicted BP level and DSST score. For each unit (mm Hg) increment in genetically-predicted BP level, there was a 0.49 to 0.67-point lower DSST score. There was no significant association between genetically-predicted BPs and performance on the RAVLT or Stroop Interference test. The direction of association was however consistent among the 3 cognitive tests (Table 2).

**Table 2:** Causal association between blood pressure and midlife cognitive function inferred by inverse-variance weighted MR analysis

	DSST			RAVLT			Stroop		
	SBP	DBP	PP	SBP	DBP	PP	SBP	DBP	PP
**IVW**									
**Estimate**^a^	− 0.49	−0.66	− 0.52	− 0.04	− 0.04	− 0.07	0.15	0.18	0.16
**95% CI**	−0.79, − 0.18	− 1.20, − 0.13	−0.99, − 0.04	−0.11, 0.03	− 0.16, 0.08	−0.18, 0.03	0.03, 0.32	−0.12, 0.48	− 0.11, 0.43
***P*-value**	0.002	0.014	0.033	0.242	0.481	0.164	0.100	0.236	0.251
**IVW after removing outliers**									
**Estimate**^a^	− 0.49	−0.77	− 0.67	− 0.07	− 0.05	−0.07	0.23	0.24	0.27
**95% CI**	−0.80, − 0.17	−1.03, − 0.23	− 1.16, − 0.18	− 0.14, 0.00	− 0.16, 0.06	−0.17, 0.03	0.05, 0.41	−0.07, 0.54	− 0.01, 0.56
***P*-value**	0.002	0.005	0.008	0.046	0.37	0.165	0.011	0.126	0.058

*Abbreviations*: *IVW* Inverse-variance weighted, *MR* Mendelian randomization, *SBP* Systolic Blood Pressure, *DBP* Diastolic Blood Pressure, *PP* Pulse Pressure, *DSST* Digit Symbol Substitution Test, *RAVLT* Rey Auditory Verbal Learning Test, *Stroop* The Stroop Interference test

^a^ Represents the change in cognitive test score per 1-unit (mmHg) change in BP

We conducted analyses to detect potential invalid and/or influential instruments. Radial plots indicated the presence of outlier instruments (Figure S2 in Additional file 1). After removing these outliers, the causal associations between genetically-predicted BPs and midlife cognitive function were generally strengthened (Table 2). Notably, genetically-predicted SBP was now also associated with RAVLT and Stroop performance. Each unit (mm Hg) increment in genetically-predicted SBP level was associated with a 0.07-point lower RAVLT score and a 0.23-point higher Stroop Interference test score (Table 2).

The IVW method is optimally efficient when all genetic variants are valid IVs but is biased when one or more genetic variants are invalid IVs. Therefore, we performed sensitivity analyses using additional MR methods that vary in their robustness to the presence of invalid IVs (Tables 3 and 4). The weighted median method indicated a similar or stronger magnitude of causal associations, although with larger confidence intervals, while the MR-Egger method was insufficiently powered. Estimates from the contamination mixture MR method were similar to those of the IVW (Tables 3 and 4). The Bowden *I*^*2*^ statistics across the three BP exposures were all high, around 0.96, indicating a high strength of IVs and a less than moderate degree of NOME assumption violation (Tables 3 and 4). The mean F-statistics, another measure of IV strength, were 29.4 for SBP, 32.8 for DBP, and 26.0 for PP, indicating that our causal estimates are not likely to be biased by weak IVs.

**Table 3:** Sensitivity analyses for robust estimation of the causal association between blood pressure and midlife cognitive function before removing outliers

	DSST			RAVLT			Stroop		
	SBP	DBP	PP	SBP	DBP	PP	SBP	DBP	PP
**Weighted Median**									
Estimate (SD)	−0.32 (0.23)	− 0.58 (0.40)	− 0.30 (0.42)	− 0.05 (0.05)	−0.06 (0.08)	0.08 (0.09)	0.19 (0.13)	0.26 (0.24)	0.08 (0.24)
*P*-value	0.154	0.15	0.462	0.341	0.484	0.352	0.146	0.269	0.731
**MR Egger Regression**									
Estimate (SD)	−0.53 (0.33)	− 0.82 (0.54)	0.19 (0.40)	0.05 (0.07)	−0.12 (0.12)	0.01 (0.09)	0.09 (0.18)	−0.12 (0.30)	−0.21 (0.23)
*P*-value	0.106	0.133	0.788	0.487	0.318	0.91	0.922	0.702	0.731
**ConMix**									
Estimate	−0.38	−0.54	−0.44	− 0.03	−0.05	− 0.01	0.17	0.28	0.18
95% CI	(−0.71, − 0.07)	(−1.03, − 0.04)	(−0.93, 0.01)	(− 0.08, 0.04)	(− 0.15, 0.07)	(−0.12, 0.08)	(0.00, 0.33)	(−0.04, 0.56)	(− 0.07, 0.45)
***I***^**2**^ **statistic**									
*P*-value	0.97	0.97	0.96	0.97	0.97	0.96	0.97	0.97	0.96
**MRPRESSO**									
*P*-value	0.83	0.49	0.61	0.06	0.09	0.11	0.51	0.49	0.36
**Cochran Statistic**									
Q Statistics	85.93	126.23	91.71	119.19	116.26	113.72	97.76	97.69	101.15
*P*-value	0.803	0.648	0.633	0.072	0.089	0.118	0.488	0.461	0.366
**Egger Intercept Test**									
Estimate (SD)	0.02 (0.14)	0.05 (0.14)	−0.22 (0.11)	−0.05 (0.03)	0.02 (0.03)	−0.03 (0.03)	0.06 (0.08)	0.09 (0.08)	0.13 (0.06)
*P*-value	0.874	0.74	0.055	0.15	0.453	0.236	0.426	0.259	0.045

*Abbreviations*: *MR* Mendelian randomization, *SBP* Systolic Blood Pressure, *DBP* Diastolic Blood Pressure, *PP* Pulse Pressure, *DSST* Digit Symbol Substitution Test, *RAVLT* Rey Auditory Verbal Learning Test, *Stroop* The Stroop Interference test, *MRPRESSO* Mendelian Randomization Pleiotropy RESidual Sum and Outlier, *conMIX* Contamination mixture, *SD* Standard deviation, *CI* Confidence Interval

**Table 4:** Sensitivity analyses for robust estimation of the causal association between blood pressure and midlife cognitive function after removing outliers

	DSST			RAVLT			Stroop		
	SBP	DBP	PP	SBP	DBP	PP	SBP	DBP	PP
**Weighted Median**									
Estimate (SD)	−0.33 (0.22)	−0.86 (0.39)	− 0.31 (0.42)	−0.09 (0.05)	− 0.06 (0.09)	0.07 (0.09)	0.21 (0.13)	0.26 (0.23)	0.25 (0.23)
*P*-value	0.141	0.027	0.451	0.066	0.512	0.442	0.114	0.252	0.272
**MR Egger Regression**									
Estimate (SD)	−0.66 (0.33)	−0.88 (0.55)	−0.26 (0.42)	− 0.05 (0.07)	−0.13 (0.12)	0.04 (0.09)	−0.08 (0.19)	− 0.24 (0.30)	−0.06 (0.24)
*P*-value	0.048	0.111	0.526	0.483	0.269	0.643	0.656	0.437	0.272
**ConMix**									
Estimate	−0.48	−0.79	−0.65	− 0.07	−0.06	− 0.07	0.2	0.25	0.28
95% CI	(−0.80, − 0.15)	(−1.36, − 0.24)	(−1.14, −.17)	(−0.13, 0.00)	(− 0.19, 0.07)	(− 0.17, 0.02)	(0.02, 0.37)	(− 0.13, 0.57)	(0.00, 0.55)
***I***^**2**^ **statistic**									
*P*-value	0.97	0.97	0.96	0.96	0.97	0.96	0.97	0.97	0.96
**MRPRESSO**									
*P*-value	0.97	0.94	0.96	0.95	0.90	0.89	0.93	0.92	0.96
**Cochran Statistic**									
Q Statistics	71.01	97.24	71.33	70.16	75.22	74.98	71.65	74.18	67.53
*P*-value	0.97	0.47	0.96	0.95	0.88	0.90	0.95	0.93	0.96
**Egger Intercept Test**									
Estimate (SD)	0.09 (0.14)	0.04 (0.14)	−0.14 (0.12)	−0.01 (0.03)	0.02 (0.03)	−0.04 (0.02)	0.16 (0.08)	0.14 (0.08)	0.12 (0.07)
*P*-value	0.553	0.805	0.222	0.766	0.444	0.105	0.057	0.073	0.082
**nSNPS**	96	93	94	92	92	93	94	94	91

*Abbreviations*: *MR* Mendelian randomization, *SBP* Systolic Blood Pressure, *DBP* Diastolic Blood Pressure, *PP* Pulse Pressure, *DSST* Digit Symbol Substitution Test, *RAVLT* Rey Auditory Verbal Learning Test, *Stroop* The Stroop Interference test, *MRPRESSO* Mendelian Randomization Pleiotropy RESidual Sum and Outlier, *conMIX* Contamination mixture, *SD* Standard deviation, *CI* Confidence Interval

We also conducted sensitivity analyses to investigate potential pleiotropy, which could invalidate MR assumptions and bias causal estimates. Indeed, a key assumption of MR is that the IVs have an effect on the outcome (cognition) only via the exposure (BP). Using the MR-Egger intercept test, we did not detect evidence of significant directional pleiotropy in any of the analyses performed. Consistently, the MR-PRESSO global test also did not indicate any evidence of directional pleiotropy (Tables 3 and 4). We also plotted IV strength against IV estimates in funnel plots (Figure S3) and confirmed the absence of pleiotropy. All of these plots showed a symmetrical distribution indicating that pleiotropic effects were well balanced across all the genetic variants. Cochran Q statistics also confirmed that there was no evidence of heterogeneity in causal estimates (Tables 3 and 4).

To further preclude the possibility that the causal inference was driven by any individual variant, we performed additional sensitivity analyses. Leave-one-out results confirmed that the causal estimates were not driven by the influence of any individual SNP (Figures S4 and S5). Indeed, while omitting some variants placed heavier burden on causal estimates than others, these estimates were, both, stable and consistent in direction (did not cross the 0 line).

## Discussion

Mendelian randomization holds substantial promise for causal inference from observational data. This approach mimics the random allocation in RCTs by using risk factor-associated genetic variants as IVs. It is based on the principle that the distribution of those genetic variants in the population is randomly assigned at meiosis, independent of potential confounders and not susceptible to reverse causation or other biases [17]. In this study, we inferred the causal effect of high BP on midlife cognitive function through a two-sample MR method and further sensitivity analyses. We report that higher genetically-predicted BP, especially SBP, is causally associated with lower midlife processing speed and lower executive function and verbal memory but the estimated effects of genetically-predicted BP on midlife cognitive function is small.

A recent MR study from the UK Biobank and including participants aged 40 to 70 years observed no detrimental effect of genetically-predicted SBP on cognitive ability measured by a verbal-numerical reasoning test [28]. Given the differences in study design, including cognitive assessment and participant characteristics, it is possible that one does not contradict the other. Indeed, compared to our study, the UK Biobank study included a large range in population age. Including older individuals into the analysis has the potential to introduce biases owing to the effect of high blood pressure on mortality [29, 30]. Moreover, the relationship between BP and cognitive function may vary across the lifespan. For example, a MR study recently reported that higher genetically-predicted SBP significantly lowered the risk of Alzheimer’s disease [31].

The specific mechanisms that underlie the causal association between high BP and poor processing speed are not known. One possible mechanism may involve the known pathologies of hypertension-induced brain vascular injury, most notably to the cerebral small vessels. Indeed, long-term hypertension is known to cause vascular hypertrophy and microvascular remodeling, which result in regional cerebral blood flow dysfunction and lead to white matter disease and neuronal loss [32]. Indeed, previous studies have suggested that elevated BP is a major risk factor for several magnetic resonance imaging (MRI) markers of cerebral small vessel disease (SVDs) [33]. For example, elevated SBP is associated with brain atrophy, reduced gray matter volume and white matter hyperintensities (WMH) [34], while DBP is associated with brain atrophy and WMH [35]. It has been shown that the presence of SVD appears to align with a compromised cognitive profile of early-impaired processing speed [36, 37]. Elevated BP may also affect brain anatomical connectivity. Indeed, regional white matter integrity is lower among individuals with higher BP, regardless of hypertension status [38]. In a recent study of cognitively healthy older adults, the relationship between SBP and poorer processing speed appeared to be mediated by functional connectivity of the right superior temporal gyrus within the ventral attention network (VAN) [39]. This is consistent with a previous report showing that variation in structural organization within the frontoparietal system, which comprises the VAN, is associated with differences in attentional functions, including visual short-term memory capacity, processing speed, and spatial bias [40].

Whether antihypertensive treatment can reverse the pathological process of vascular damage, restore cerebral function, and improve cognitive ability remains inconclusive. On one hand, it appears that lowering BP is helpful to rehabilitate some of the vascular functions impaired by BP elevation. For instance, a previous clinical trial among subjects older than 70 suggested that right-shifted cerebral autoregulation curve induced by hypertension can be back-shifted towards normal through intensive BP lowering [41]. On the other hand, however, most clinical trials have failed to identify significant benefits of antihypertensive therapy in improving cognitive fitness [8]. The majority of these studies were carried out over a short period of time and limited to aged populations on average between 62 to 83 years of age. Elderly individuals may be exposed to the negative effects of hypertension for a longer time and may have more severe vascular injury due to hypertension than middle-aged adults, and, thus, may be more resistant to the benefits of BP lowering. Moreover, the effects of antihypertensive treatment on cognitive function or risk of dementia have been shown to differ across therapeutic classes [42], suggesting that the neuroprotective effects of antihypertensive therapy may extend beyond BP lowering.

Strengths of our study include the use of a multi-SNP genetic instrument with well-established associations with BP. The largest BP GWAS at the time of this analysis reported genetic association for 267 SNPs in total, 115 of which were newly validated [16]. We opted for the smaller set of SNPs, which were previously identified with stronger evidence of validity, although fewer IVs theoretically reduce statistical power [43]. The use of summary data for gene-BP association from a large GWAS analysis also improved power and precision of our study. However, several limitations are acknowledged. First, the genetic variants for the various BP measures almost fully overlapped, which makes it difficult to discriminate possible unique causal relationships between the specific components of BP and cognitive function. Excluding genetic variants that overlap among BP traits would likely result in a loss of strength of our MR analyses. Second, possible effects of unmeasured confounders are a recognized limitation of the MR approach. In particular, the possible confounding effects of antihypertensive medication use could not be accounted for in our analyses because the estimates of allelic effects on BP were obtained from a GWAS that corrected for medication use by adding 15 and 10 mmHg to the SBP and DBP values, respectively. If genetically predicted higher BP is also associated with a higher probability of being on antihypertensive medication as previously observed [31], the true causal association between BP and cognitive function is likely underestimated. Moreover, the GWAS summary statistics used in this analysis adjusted for BMI and, thus, can potentially yield biased estimates. However, a recent study using both simulations and empirical data from the UK Biobank showed that using adjusted and corrected GWAS in MR analysis is unlikely to make a large difference to causal estimates [44]. Third, these analyses were conducted in persons of European ancestry. Whether the results are generalizable to diverse populations remains to be examined. Expanding efforts for genetic discoveries of BP in African-Americans and Hispanics/Latinos is required to meet this need. Finally, although this study provided evidence for a causal relationship between SBP and processing speed, it does not shed light on the possible biological mechanisms involved.

## Conclusion

By providing support for a causal relationship between BP and cognitive health in middle age, our MR study underscores the need for further investigations of the role and mechanisms of BP dysfunction on cognitive health across the lifespan, which may inform on early intervention and timely treatment of hypertension to maintain brain health.

## Supplementary information

**Additional file 1: **Cognitive assessment in CARDIA at Year 25 examination (2010–2011); **Table S1.** SNPs and SNP-exposure effects used in the MR analyses.; **Figure S1.** Summary of the MR approach used in the study. **Figure S2.** Radial plot for detection of outliers. **Figure S3.** Funnel plot of blood pressure effect on cognitive function. **Figure S4.** Leave-one-out result of IVW estimates. **Figure S5.** Leave-one-out result of MR-Egger estimates.

## Data Availability

CARDIA data are available through the National Center for Biotechnology Information database of Genotypes and Phenotypes (dbGaP). More information is provided at https://www.cardia.dopm.uab.edu/study-information/genetic-data/cardia-genetic-data-in-dbgap-ncbi.

## Abbreviations

BP: Blood Pressure
BMI: Body Mass Index
CARDIA: Coronary Artery Risk Development in Young Adults
DBP: Diastolic Blood Pressure
DSST: Digit Symbol Substitution Test
GWAS: Genome-wide Association Study
IV: Instrumental Variable
IVW: Inverse-Variance Weighted
LD: Linkage Disequilibrium
MCI: Mild Cognitive Impairment
MR: Mendelian Randomization
NOME: NO Measurement Error
PP: Pulse Pressure
RAVLT: Rey Auditory Verbal Learning Test
RCT: Randomized Controlled Trial
SBP: Systolic Blood Pressure
SD: Standard Deviation
SNP: Single Nucleotide Polymorphism
SVD: Cerebral Small Vessel Disease
VAN: Ventral Attention Network
WMH: White Matter Hyperintensities
