# Supplementary material for: Association of blood pressure with cognitive function at midlife: a Mendelian randomization study
Source: BMC Med Genomics. 2020 Aug 26;13:121. doi: 10.1186/s12920-020-00769-y (PMC7448985; doi:10.1186/s12920-020-00769-y)
Supplement: Supplementary file 1 — Additional file 1: Cognitive assessment in CARDIA at Year 25 examination (2010–2011); Table S1. SNPs and SNP-exposure effects used in the MR analyses.; Figure S1. Summary of the MR approach used in the study. Figure S2. Radial plot for detection of outliers. Figure S3. Funnel plot of blood pressure effect on cognitive function. Figure S4. Leave-one-out result of IVW estimates. Figure S5. Leave-one-out result of MR-Egger estimates. [file 12920_2020_769_MOESM1_ESM.docx]

**Association of Blood Pressure with Cognitive Function at Midlife: A Mendelian Randomization Study – Additional File 1**

**Cognitive assessment in CARDIA at Year 25 examination (2010-2011)**

Three cognitive function tests were administered at Year 25 visit. They are:

(1) The Rey Auditory Verbal Learning Test (RAVLT): a measure of verbal learning and memory. The abilities of immediate recall, short-term episodic memory, and long-delay (10 minutes) free recall were assessed by the number of words recalled from a 15‐word list as three separate scores. Tests were consisted of five presentations of List A with free recall, one presentation of a 15-word interference list (List B), one short delay free recall of List A, and one long 10 min delay free recall of List A. We calculated a composite score by averaging the three separately measured scores. Test results from the long-delay (10 minutes) free recall were used in primary analyses. The long-delayed test score ranges from 0 to 15, with higher score indicating better performance; (2) the Digit Symbol Substitution Test (Part D), a measure primarily of psychomotor speed, as well as sustained attention and working memory (referred to as processing speed). The range of digit counts correctly substituted by symbols ranges from 0 to 133, with more correct digits indicating better performance. (3) the Stroop Interference Test, a measure of executive function. It evaluates the ability to view complex visual stimuli and respond to one stimulus dimension while suppressing the response to another dimension. It consists of three subtests, each of which is scored by counting the seconds it takes the participants to read words printed in a different color ink plus the number of errors. An interference score was calculated by subtracting the score on the third test from that on the second test. It ranges from 1 to 160, with larger score indicating poor performance.

**Table S1.** SNPs and SNP-exposure effects used in the MR analyses. Beta and standard error were derived from the large BP GWAS of Warren et al.

| **SNP** | **Effect Allele** | **Other Allele** | **SBP.beta** | **SBP.se** | **DBP.beta** | **DBP.se** | **PP.beta** | **PP.se** |
| --- | --- | --- | --- | --- | --- | --- | --- | --- |
| rs10164833 | G | C | 0.02 | 0.09 | 0.05 | 0.05 | -0.04 | 0.06 |
| rs10224002 | G | A | 0.42 | 0.08 | 0.22 | 0.04 | 0.21 | 0.05 |
| rs1036477 | G | A | -0.61 | 0.12 | 0.06 | 0.07 | -0.68 | 0.08 |
| rs10760117 | G | T | -0.21 | 0.07 | -0.07 | 0.04 |  |  |
| rs10943605 | A | G | 0.36 | 0.07 | 0.26 | 0.04 | 0.10 | 0.05 |
| rs10948071 | T | C |  |  | -0.10 | 0.04 |  |  |
| rs111245230 | C | T | 0.87 | 0.19 | 0.41 | 0.11 | 0.46 | 0.13 |
| rs11222084 | T | A | 0.39 | 0.07 | -0.23 | 0.04 | 0.62 | 0.05 |
| rs11229457 | T | C | -0.47 | 0.09 |  |  | -0.29 | 0.06 |
| rs1126464 | C | G | 0.07 | 0.08 | 0.15 | 0.05 | -0.08 | 0.06 |
| rs11556924 | T | C | -0.18 | 0.07 | -0.21 | 0.04 | 0.03 | 0.05 |
| rs11639856 | A | T | -0.39 | 0.09 |  |  | -0.23 | 0.06 |
| rs1173771 | G | A | 0.74 | 0.07 | 0.32 | 0.04 |  |  |
| rs12521868 | T | G | 0.04 | 0.07 | -0.09 | 0.04 | 0.13 | 0.05 |
| rs1275988 | T | C | -0.55 | 0.07 | -0.27 | 0.04 | -0.28 | 0.05 |
| rs12940887 | T | C |  |  | 0.18 | 0.04 |  |  |
| rs12946454 | T | A | 0.43 | 0.08 | 0.19 | 0.04 | 0.24 | 0.05 |
| rs12958173 | C | A | -0.33 | 0.08 | -0.18 | 0.04 | -0.15 | 0.05 |
| rs13082711 | C | T | 0.27 | 0.08 | 0.17 | 0.05 | 0.10 | 0.06 |
| rs13139571 | A | C | -0.41 | 0.08 | -0.31 | 0.05 | -0.10 | 0.06 |
| rs13209747 | T | C | 0.59 | 0.07 | 0.33 | 0.04 | 0.26 | 0.05 |
| rs1327235 | G | A | 0.36 | 0.07 | 0.26 | 0.04 | 0.10 | 0.05 |
| rs13333226 | G | A |  |  | -0.35 | 0.05 |  |  |
| rs13359291 | A | G | 0.46 | 0.10 | 0.29 | 0.05 | 0.16 | 0.07 |
| rs1344653 | G | A | 0.34 | 0.07 | -0.05 | 0.04 | 0.39 | 0.05 |
| rs1378942 | A | C | -0.54 | 0.07 | -0.43 | 0.04 | -0.10 | 0.05 |
| rs1421811 | G | C |  |  |  |  | -0.43 | 0.05 |
| rs1458038 | T | C | 0.99 | 0.08 | 0.56 | 0.04 | 0.43 | 0.05 |
| rs1530440 | T | C | -0.69 | 0.09 | -0.46 | 0.05 | -0.23 | 0.06 |
| rs1563788 | T | C | 0.31 | 0.08 |  |  | 0.20 | 0.05 |
| rs167479 | T | G | -0.68 | 0.07 | -0.39 | 0.04 | -0.29 | 0.05 |
| rs16823124 | A | G | 0.17 | 0.08 | 0.25 | 0.04 | -0.07 | 0.05 |
| rs16851397 | G | A | 0.45 | 0.17 | 0.33 | 0.09 | 0.12 | 0.11 |
| rs17010957 | C | T | 0.32 | 0.10 | 0.06 | 0.06 | 0.26 | 0.07 |
| rs17030613 | C | A | 0.52 | 0.09 | 0.41 | 0.05 | 0.10 | 0.06 |
| rs17080102 | C | G | -0.79 | 0.14 | -0.51 | 0.08 | -0.28 | 0.09 |
| rs17249754 | A | G | -0.84 | 0.09 | -0.39 | 0.05 | -0.45 | 0.06 |
| rs17367504 | G | A | -1.09 | 0.09 | -0.58 | 0.05 | -0.52 | 0.06 |
| rs17428471 | T | G | 0.51 | 0.13 |  |  | 0.31 | 0.09 |
| rs17477177 | C | T | 0.91 | 0.09 | -0.08 | 0.05 | 0.99 | 0.06 |
| rs17608766 | C | T | 0.82 | 0.10 |  |  | 0.63 | 0.07 |
| rs1799945 | G | C | 0.52 | 0.10 | 0.41 | 0.05 |  |  |
| rs1813353 | C | T | -0.65 | 0.07 | -0.35 | 0.04 | -0.29 | 0.05 |
| rs1925153 | T | C | -0.15 | 0.07 | 0.09 | 0.04 | -0.24 | 0.05 |
| rs1953126 | C | T |  |  |  |  | -0.18 | 0.05 |
| rs1975487 | G | A | 0.15 | 0.07 | 0.09 | 0.04 | 0.06 | 0.05 |
| rs2004776 | T | C | 0.38 | 0.08 | 0.23 | 0.05 | 0.15 | 0.06 |
| rs2014408 | T | C |  |  | 0.30 | 0.05 |  |  |
| rs2071518 | T | C | 0.37 | 0.08 | -0.20 | 0.04 | 0.57 | 0.05 |
| rs2107595 | A | G | 0.48 | 0.10 |  |  | 0.56 | 0.07 |
| rs2282978 | C | T | -0.45 | 0.07 | 0.00 | 0.04 | -0.45 | 0.05 |
| rs2291435 | T | C | -0.30 | 0.07 | -0.11 | 0.04 | -0.19 | 0.05 |
| rs2302061 | C | G | 0.23 | 0.11 | -0.05 | 0.06 | 0.28 | 0.07 |
| rs2446848 | C | T | 0.40 | 0.19 | 0.11 | 0.11 | 0.29 | 0.13 |
| rs2493292 | T | C | 0.30 | 0.10 | 0.19 | 0.06 | 0.12 | 0.07 |
| rs2521501 | T | A | 0.74 | 0.08 | 0.37 | 0.04 | 0.36 | 0.05 |
| rs2782980 | C | T | 0.36 | 0.08 | 0.31 | 0.04 | 0.06 | 0.05 |
| rs2898290 | C | T | -0.36 | 0.07 | -0.17 | 0.04 | -0.19 | 0.05 |
| rs2969070 | A | G | -0.27 | 0.07 | -0.15 | 0.04 | -0.12 | 0.05 |
| rs3184504 | C | T | -0.60 | 0.07 | -0.53 | 0.04 | -0.07 | 0.05 |
| rs33063 | G | A | -0.25 | 0.10 | 0.04 | 0.05 | -0.29 | 0.07 |
| rs34591516 | T | C | 0.53 | 0.17 | 0.37 | 0.09 | 0.16 | 0.11 |
| rs347591 | T | G | 0.37 | 0.07 | 0.15 | 0.04 | 0.23 | 0.05 |
| rs35444 | G | A | -0.53 | 0.07 | -0.27 | 0.04 | -0.26 | 0.05 |
| rs3741378 | T | C | -0.43 | 0.10 | -0.23 | 0.06 | -0.20 | 0.07 |
| rs3752728 | A | G | 0.37 | 0.08 | 0.41 | 0.04 | -0.04 | 0.05 |
| rs3774372 | C | T | -0.43 | 0.10 | 0.32 | 0.05 | -0.75 | 0.07 |
| rs3918226 | T | C | 0.87 | 0.13 | 0.67 | 0.07 | 0.19 | 0.09 |
| rs409558 | C | T |  |  |  |  | -0.41 | 0.07 |
| rs419076 | C | T | -0.44 | 0.07 | -0.32 | 0.04 | -0.13 | 0.05 |
| rs4245739 | A | C | 0.07 | 0.08 | 0.14 | 0.04 | -0.06 | 0.05 |
| rs4494250 | A | G |  |  | 0.22 | 0.04 |  |  |
| rs452036 | A | G | -0.21 | 0.07 | 0.14 | 0.04 | -0.35 | 0.05 |
| rs4530754 | A | G | -0.07 | 0.07 | 0.11 | 0.04 | -0.18 | 0.05 |
| rs470113 | G | A | 0.34 | 0.09 | 0.07 | 0.05 | 0.27 | 0.06 |
| rs4728142 | A | G | -0.12 | 0.07 | -0.05 | 0.04 | -0.07 | 0.05 |
| rs4746172 | T | C | -0.22 | 0.08 | -0.08 | 0.04 | -0.14 | 0.05 |
| rs4823006 | G | A | -0.20 | 0.07 | -0.09 | 0.04 | -0.11 | 0.05 |
| rs6015450 | G | A | 0.76 | 0.11 | 0.61 | 0.06 | 0.14 | 0.07 |
| rs6095241 | A | G | -0.25 | 0.07 | -0.20 | 0.04 | -0.05 | 0.05 |
| rs6271 | T | C | -0.62 | 0.13 | -0.50 | 0.07 | -0.12 | 0.09 |
| rs633185 | C | G | 0.74 | 0.08 | 0.43 | 0.04 | 0.32 | 0.05 |
| rs661348 | C | T | 0.54 | 0.07 | 0.25 | 0.04 | 0.29 | 0.05 |
| rs6712094 | G | A | -0.65 | 0.08 | -0.29 | 0.04 | -0.36 | 0.05 |
| rs6722745 | C | T | 0.02 | 0.08 | 0.03 | 0.04 | -0.01 | 0.05 |
| rs6797587 | G | A | 0.39 | 0.07 | 0.26 | 0.04 | 0.13 | 0.05 |
| rs6825911 | T | C | -0.44 | 0.09 | -0.27 | 0.05 | -0.18 | 0.06 |
| rs687621 | G | A | -0.05 | 0.08 | -0.18 | 0.04 | 0.13 | 0.05 |
| rs6969780 | C | G |  |  | 0.23 | 0.07 |  |  |
| rs709209 | G | A | -0.15 | 0.07 | 0.07 | 0.04 | -0.22 | 0.05 |
| rs7103648 | G | A | 0.43 | 0.07 | 0.28 | 0.04 | 0.14 | 0.05 |
| rs7248104 | A | G | -0.32 | 0.07 | -0.08 | 0.04 | -0.24 | 0.05 |
| rs7297416 | C | A | -0.47 | 0.08 | -0.21 | 0.04 | -0.26 | 0.05 |
| rs7302981 | G | A | -0.31 | 0.07 | -0.23 | 0.04 | -0.08 | 0.05 |
| rs7406910 | C | T | 0.32 | 0.12 | 0.10 | 0.07 | 0.22 | 0.08 |
| rs7515635 | C | T | -0.26 | 0.07 | -0.13 | 0.04 | -0.13 | 0.05 |
| rs751984 | C | T |  |  | -0.46 | 0.06 |  |  |
| rs757081 | G | C | 0.45 | 0.07 |  |  | 0.30 | 0.05 |
| rs76452347 | T | C | -0.32 | 0.09 | -0.24 | 0.05 | -0.08 | 0.06 |
| rs8068318 | T | C | 0.35 | 0.08 | 0.15 | 0.04 | 0.20 | 0.05 |
| rs867186 | G | A | 0.00 | 0.12 | -0.14 | 0.07 | 0.14 | 0.08 |
| rs871606 | C | T | -0.29 | 0.11 | 0.30 | 0.06 | -0.59 | 0.08 |
| rs900145 | T | C | 0.18 | 0.08 | 0.18 | 0.04 | 0.00 | 0.05 |
| rs918466 | A | G | -0.05 | 0.07 | -0.16 | 0.04 | 0.11 | 0.05 |
| rs926552 | A | G | -0.03 | 0.10 | -0.30 | 0.06 |  |  |
| rs9306160 | C | T | 0.27 | 0.07 | 0.17 | 0.04 | 0.10 | 0.05 |
| rs932764 | G | A | 0.42 | 0.07 |  |  | 0.23 | 0.05 |
| rs9349379 | G | A | -0.30 | 0.07 | 0.06 | 0.04 | -0.36 | 0.05 |
| rs9687065 | G | A | -0.46 | 0.09 | -0.30 | 0.05 | -0.16 | 0.06 |

**Figure S1.** Summary of the MR approach used in the study


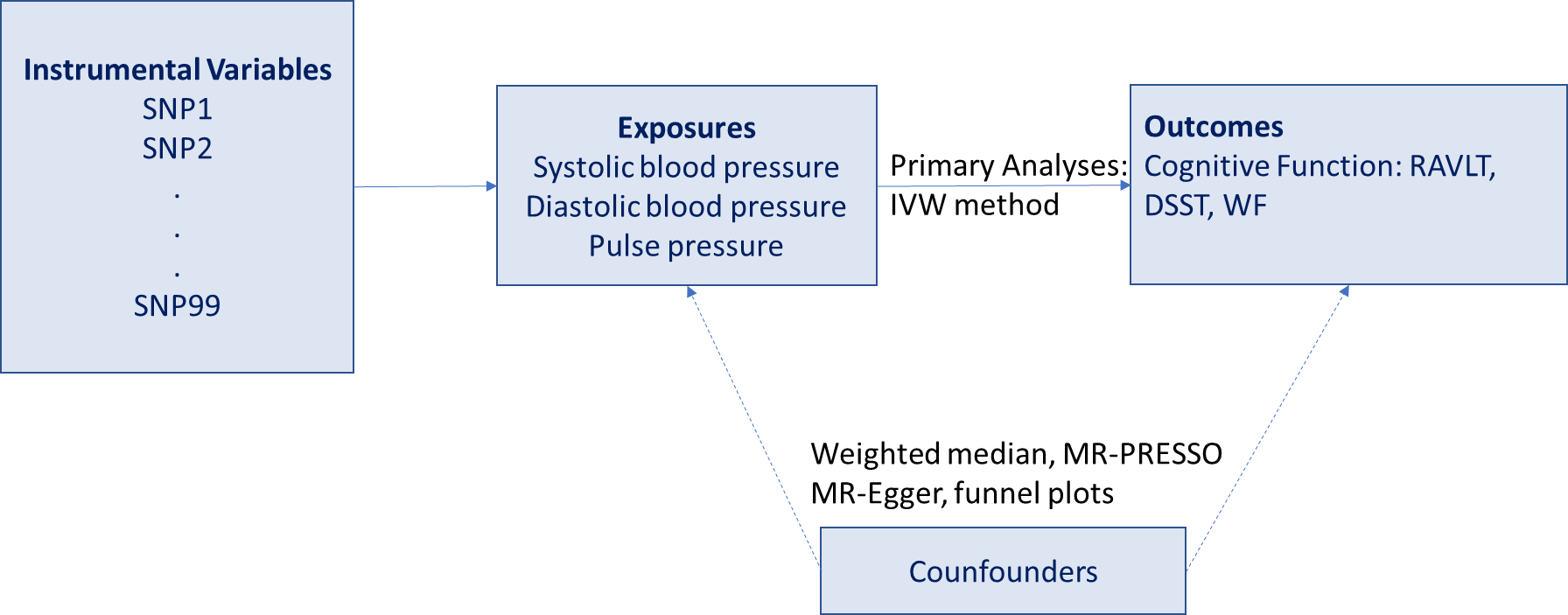


**Figure S2.** Radial plot showing outliers in the estimates of blood pressure effects on midlife cognitive function produced by *RadialMR*. (Orange line: MR-Egger estimate, blue line: Radial IVW estimate, pink dots: IVW and MR-Egger outlier, green dot: IVW outlier, yellow dot: MR-Egger outlier, black dot: SNP)

| 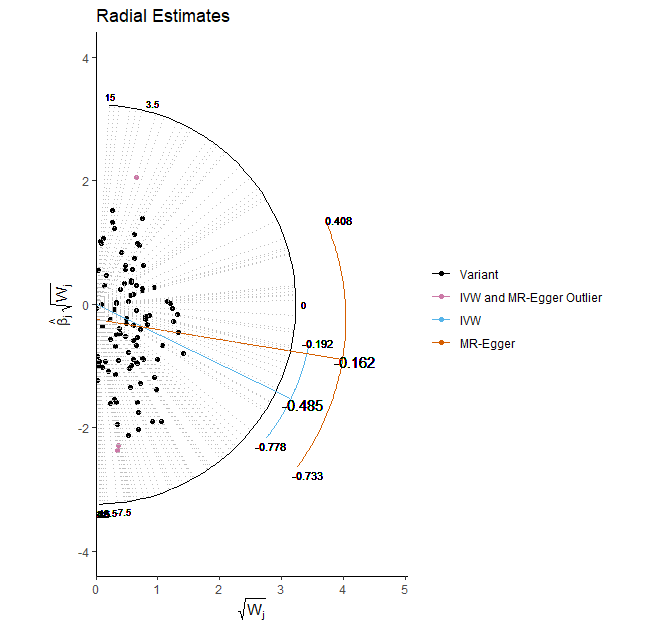Radial plot for SBP effect on DSST | Radial plot for SBP effect on RAVLT  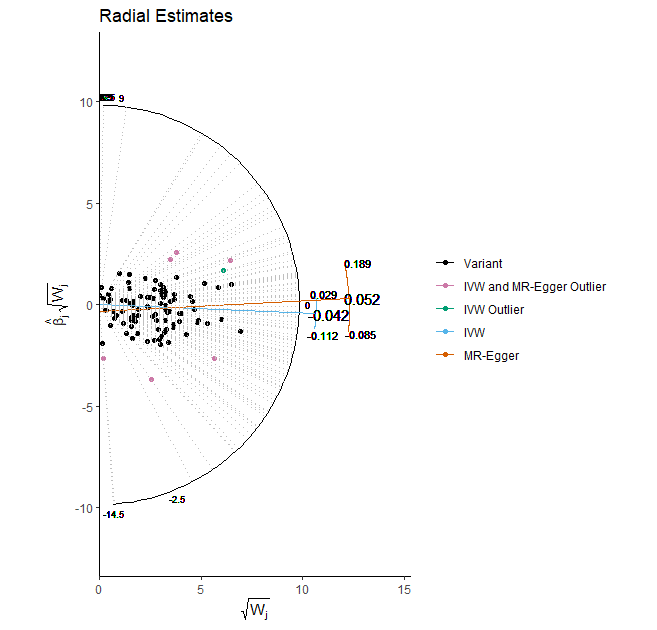 | Radial plot for SBP effect on STROOP  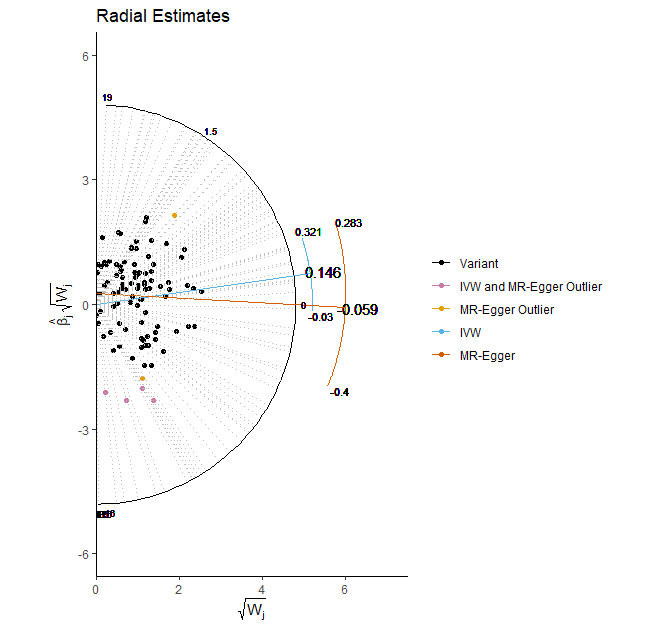 |
| --- | --- | --- |
| Radial plot for DBP effect on DSST  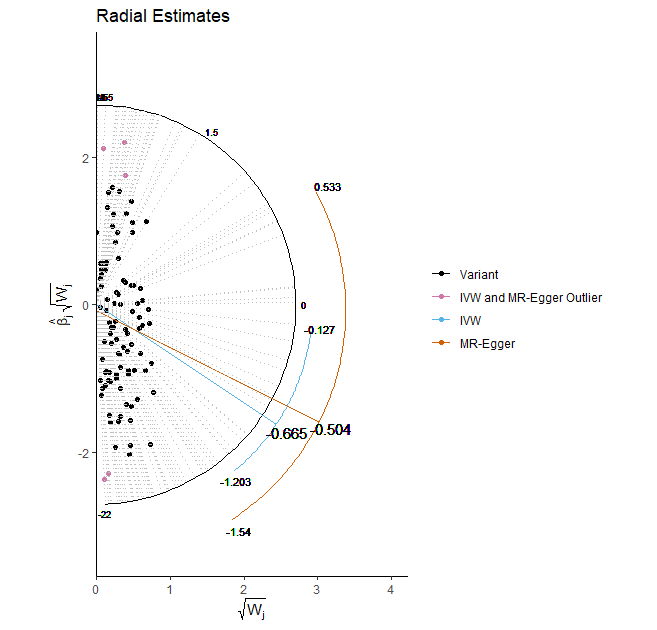 | Radial plot for DBP effect on RAVLT  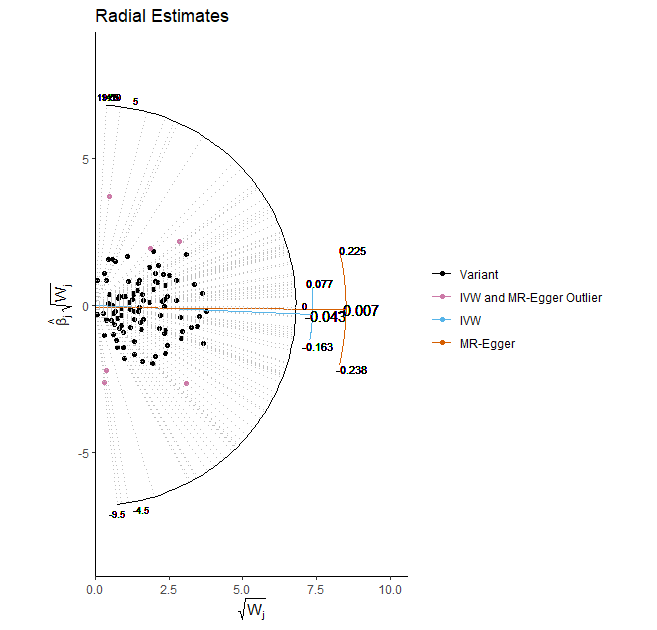 | Radial plot for DBP effect on STROOP  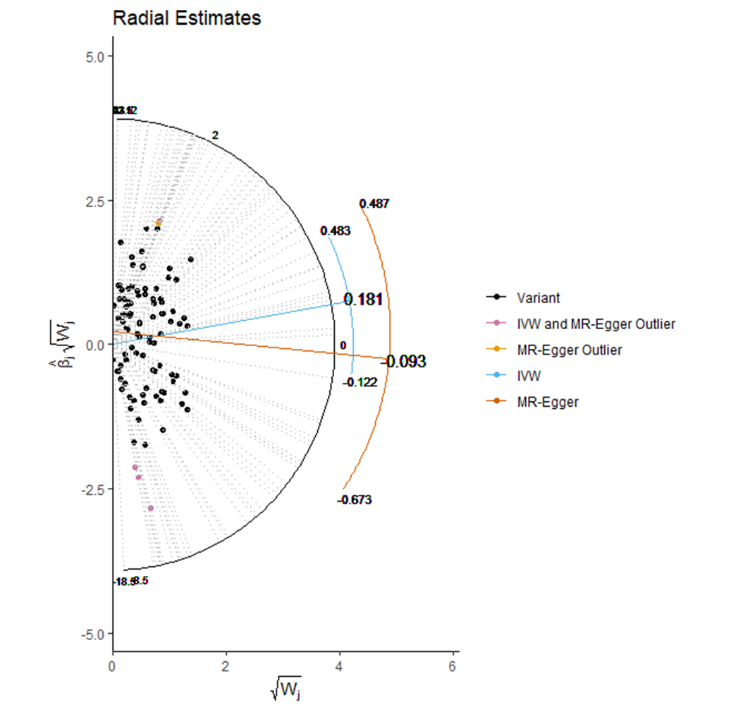 |

| Radial plot for PP effect on DSST  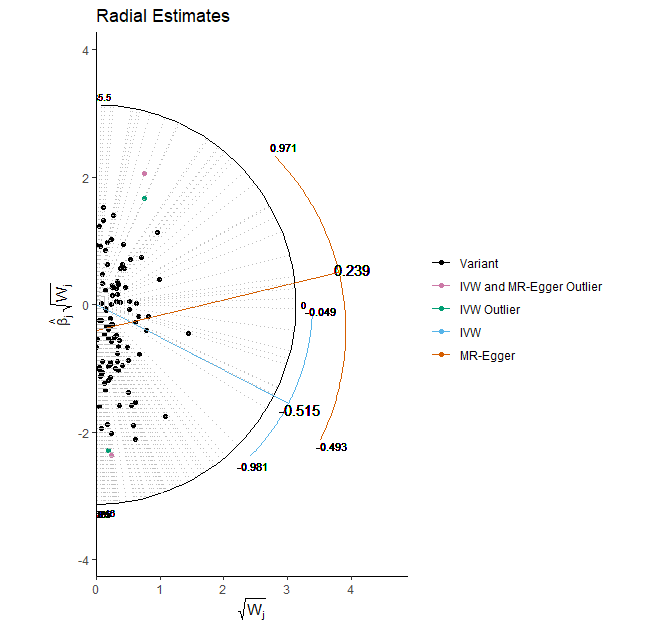 | Radial plot for PP effect on RAVLT  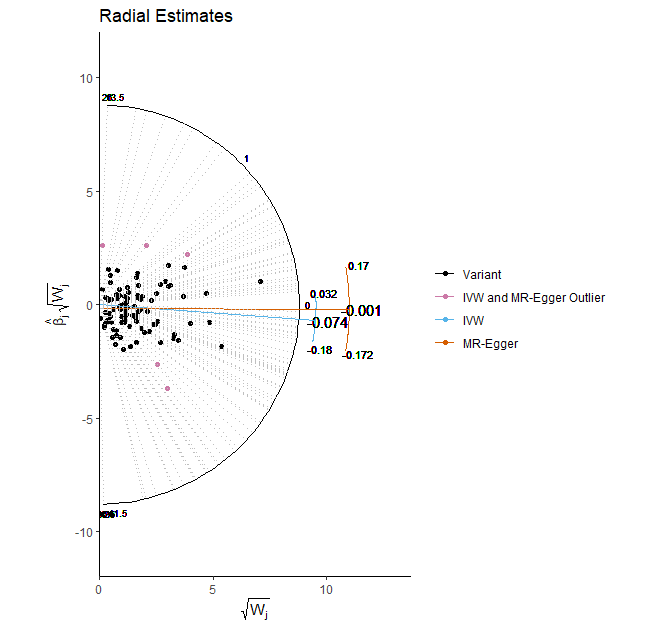 | Radial plot for PP effect on STROOP  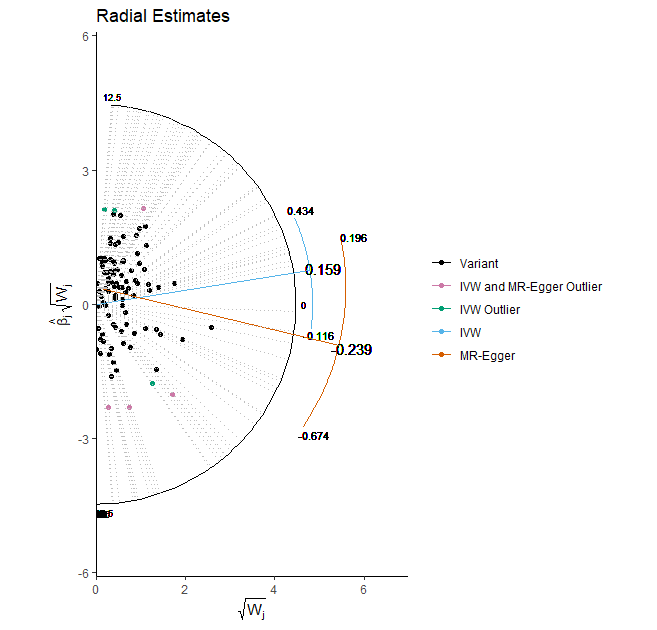 |
| --- | --- | --- |

**Figure S3.** Funnel plot of blood pressure effect on cognitive function

By displaying the instrument strength against the causal estimate derived by each selected single nucleotide polymorphisms (SNP), the funnel plots can be used as a graphical tool to visually inspect any asymmetric contribution of individual variant to the global causal inference. Symmetric funnel plots as shown in this figure suggest that only few SNPs are apparently of high strength and the individual causal estimate for each of them is closely akin to the global causal inference. The majority of these genetic variants have a relatively intermediate or low instrument strength and are symmetrically distributed around the global causal estimate, indicating that none have evidently biased the result. DSST stands for Digit Symbol Substitution Test; RAVLT, long delayed free recall of Rey Auditory Verbal Learning Test; Stroop, the Stroop Interference test.

| 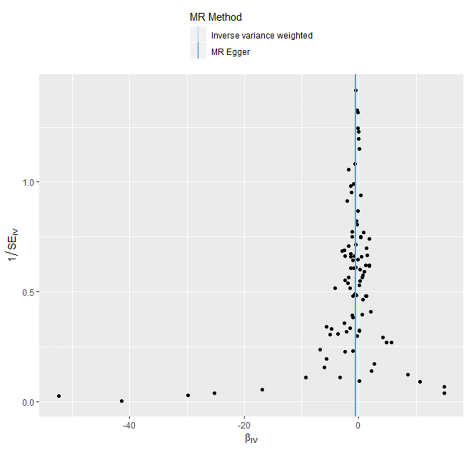 | 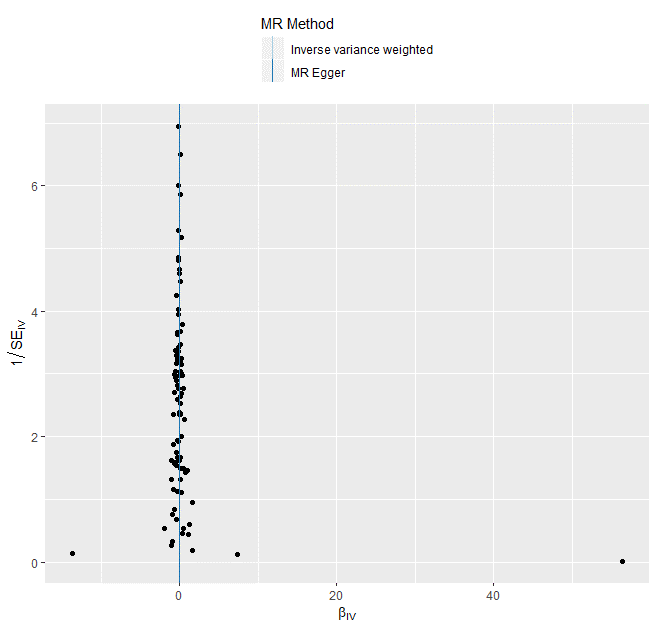 | 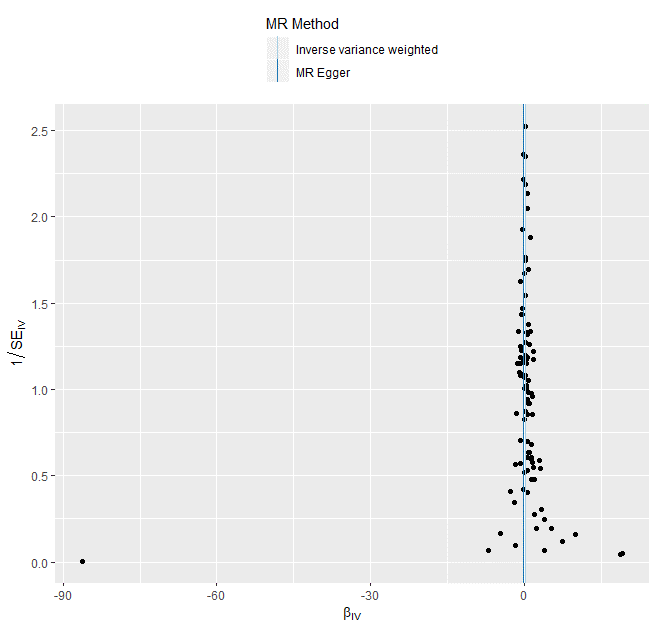 |
| --- | --- | --- |
| Funnel plot for SBP effect on DSST | Funnel plot for SBP effect on RAVLT | Funnel plot for SBP effect on STROOP |
| 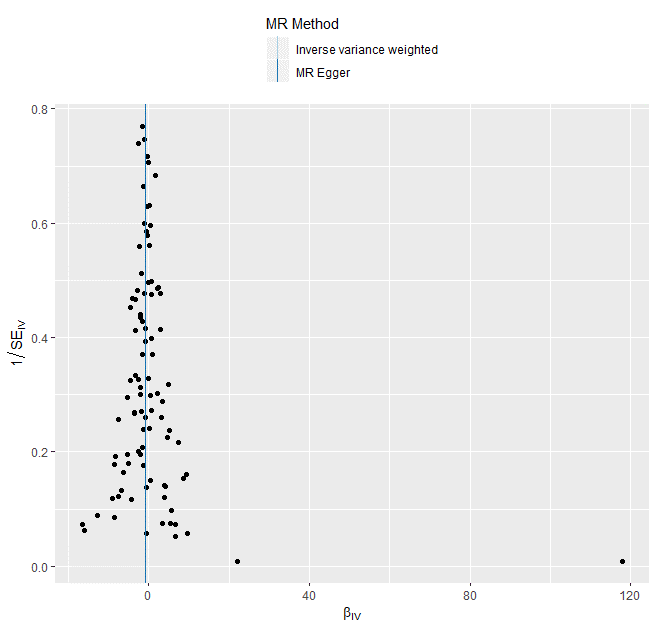 | 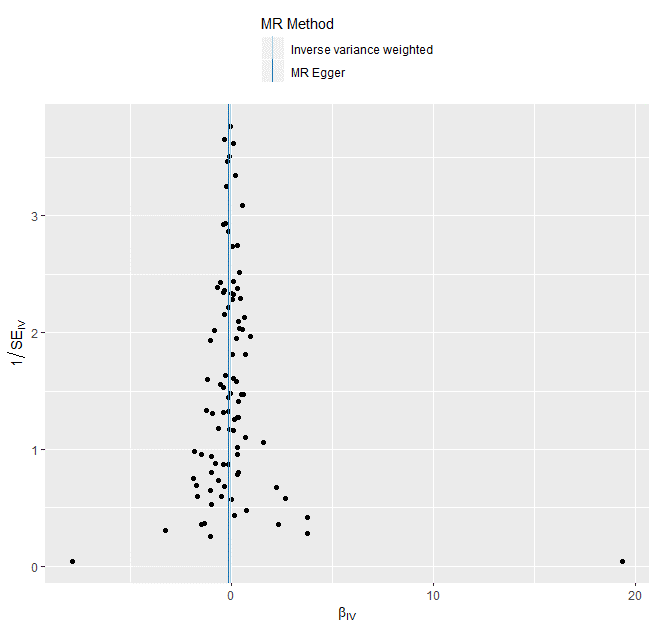 | 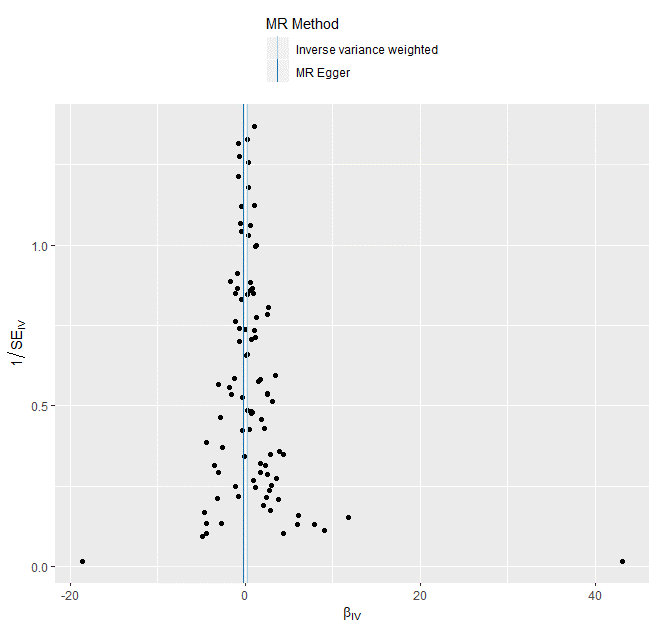 |
| Funnel plot for DBP effect on DSST | Funnel plot for DBP effect on RAVLT | Funnel plot for DBP effect on STROOP |
| 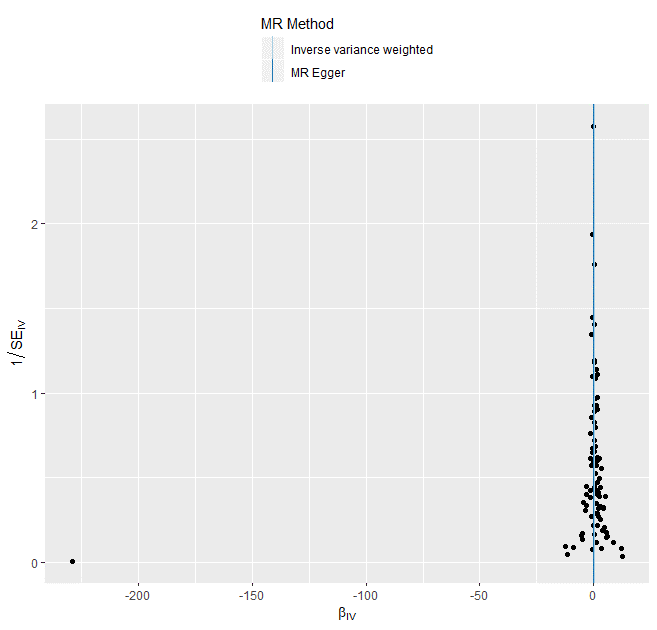 | 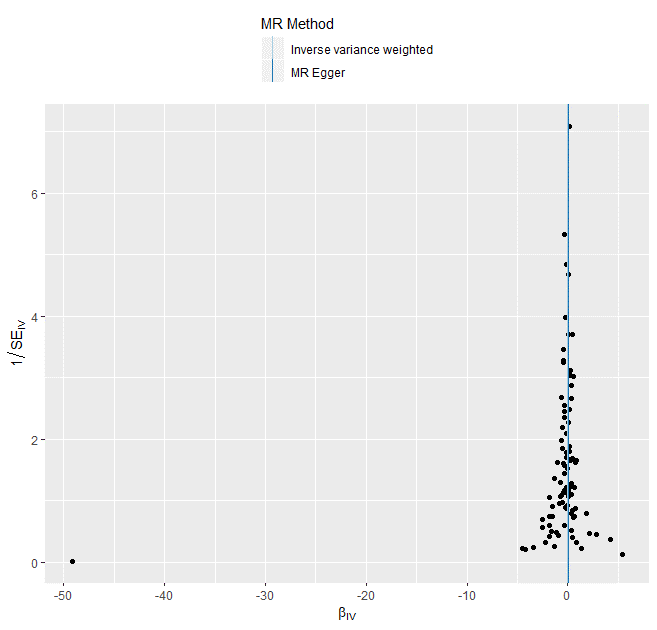 | 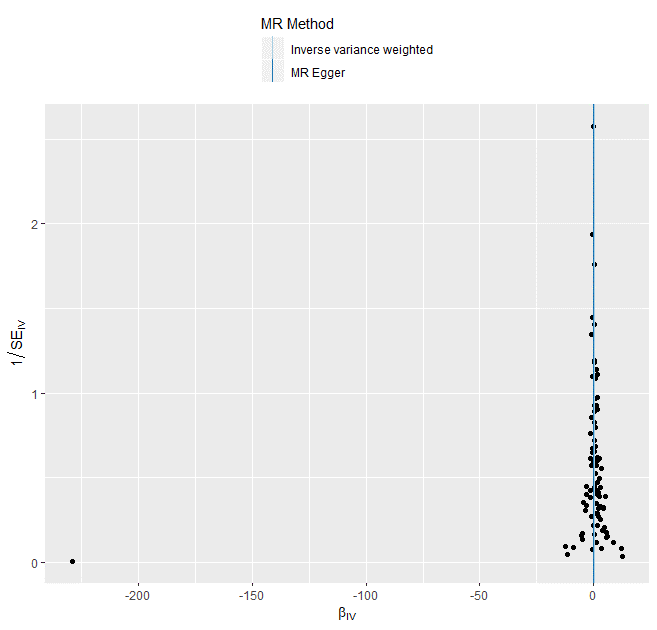 |
| Funnel plot for PP effect on DSST | Funnel plot for PP effect on RAVLT | Funnel plot for PP effect on STROOP |

**Figure S4.** Leave-one-out result of IVW estimates

By removing a single genetic variant at a time, leave-one-out results estimate the magnitude of influence from each selected genetic variant on the global causal estimate. The results showed below suggest that whichever variant is removed, the remaining pool of instruments detect a similar causal effect size as the global causal inference. All the causal estimates are in the same direction. None of them cross the horizontal zero line.

| 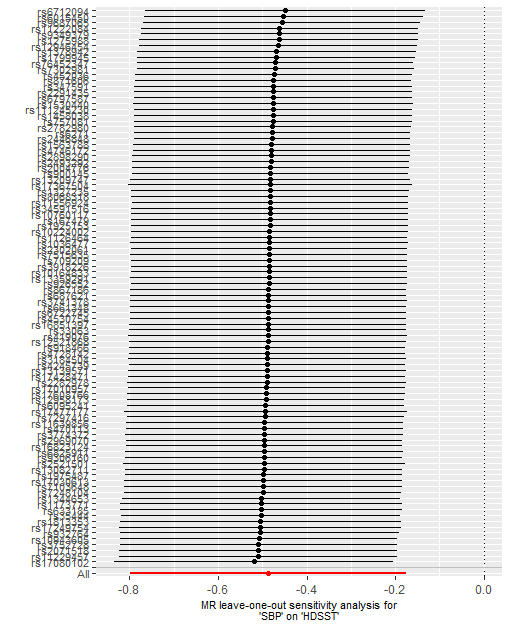 | 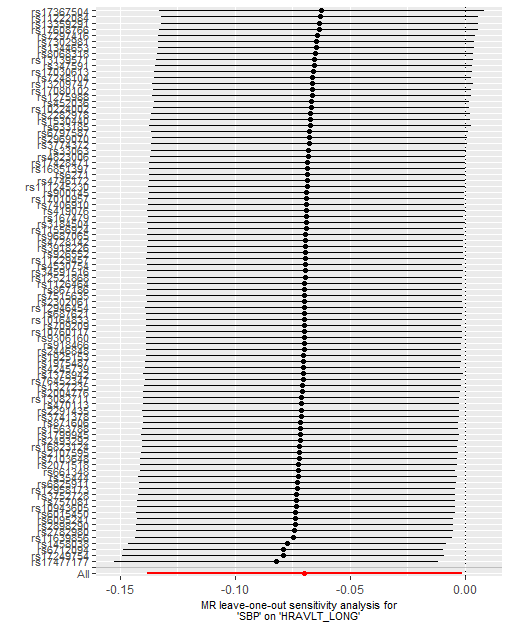 | 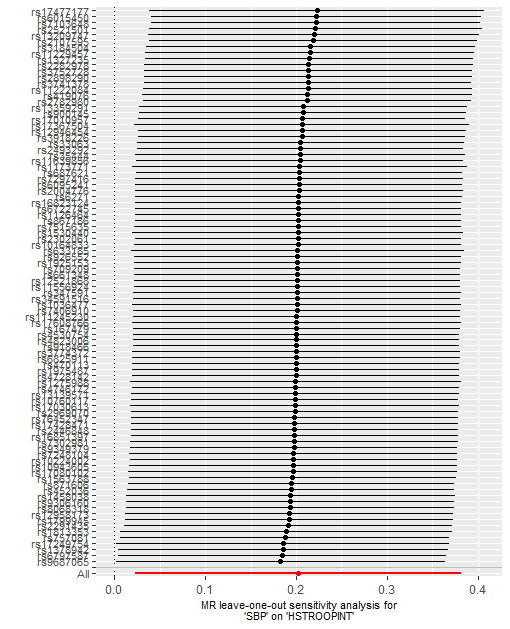 |
| --- | --- | --- |
| Leave-one-out plot for SBP effect on DSST | Leave-one-out plot for SBP effect on RAVLT | Leave-one-out plot for SBP effect on STROOP |
| 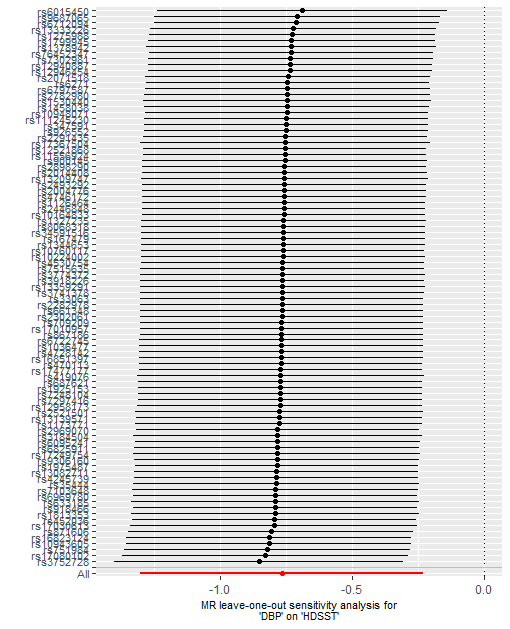 | 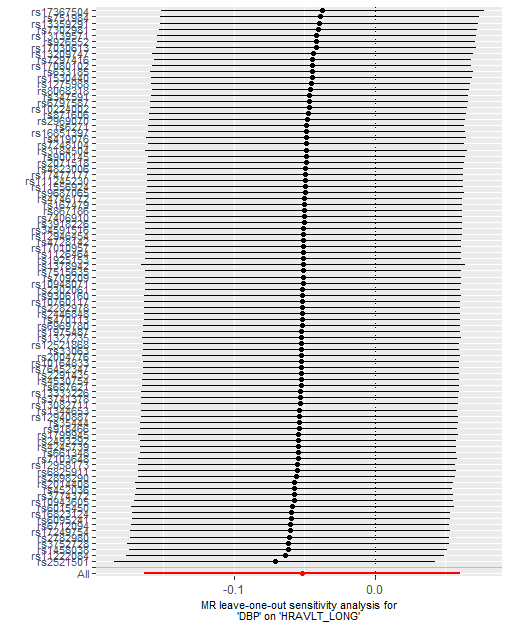 | 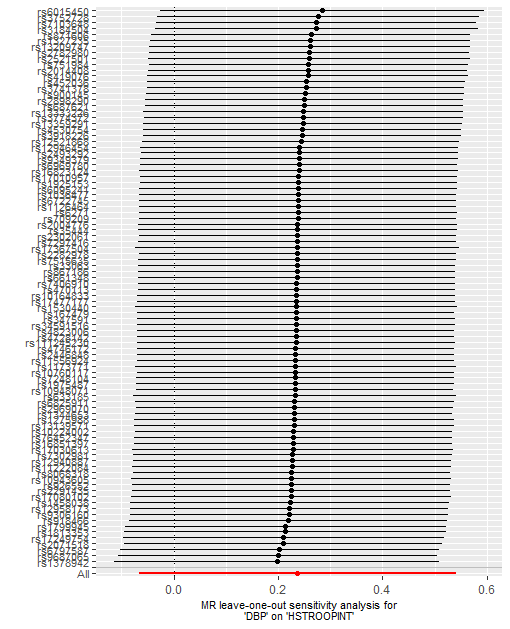 |
| Leave-one-out plot for DBP effect on DSST | Leave-one-out plot for DBP effect on RAVLT | Leave-one-out plot for DBP effect on STROOP |
| 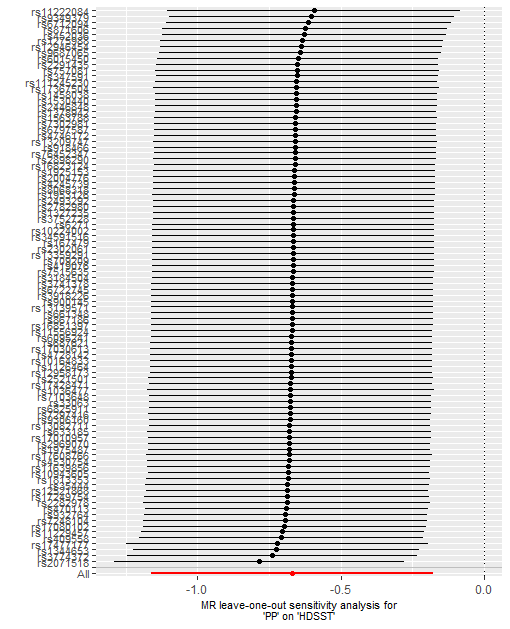 | 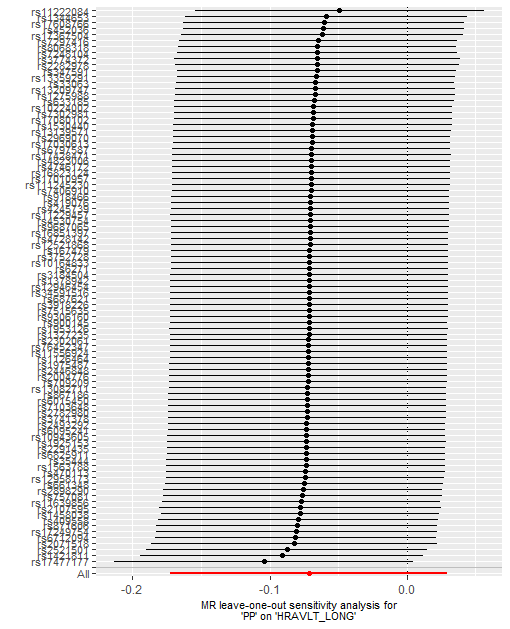 | 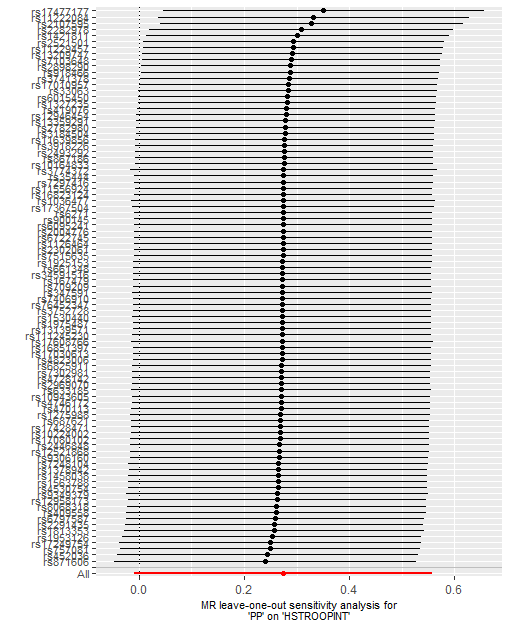 |
| Leave-one-out plot for PP effect on DSST | Leave-one-out plot for PP effect on RAVLT | Leave-one-out plot for PP effect on STROOP |
|  |  |  |

**Figure S5.** Leave-one-out result of MR-Egger estimates

| 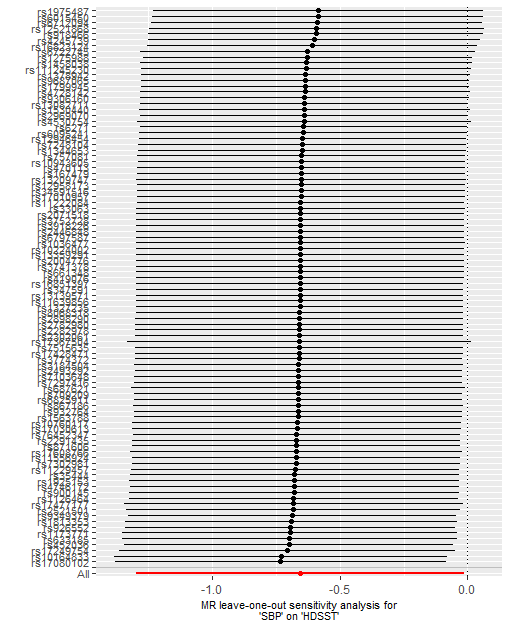 | 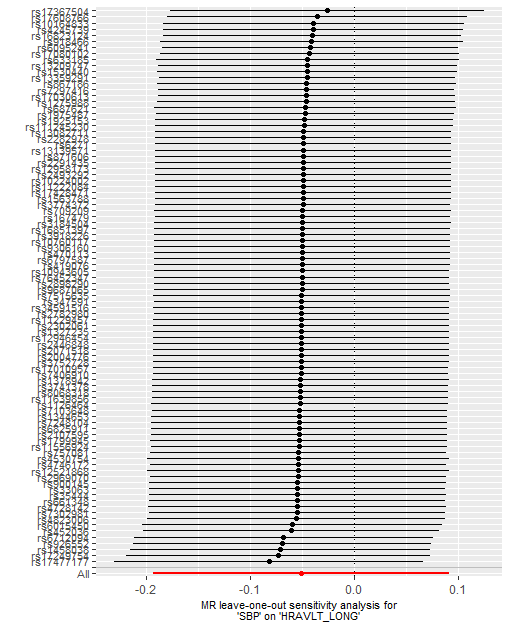 | 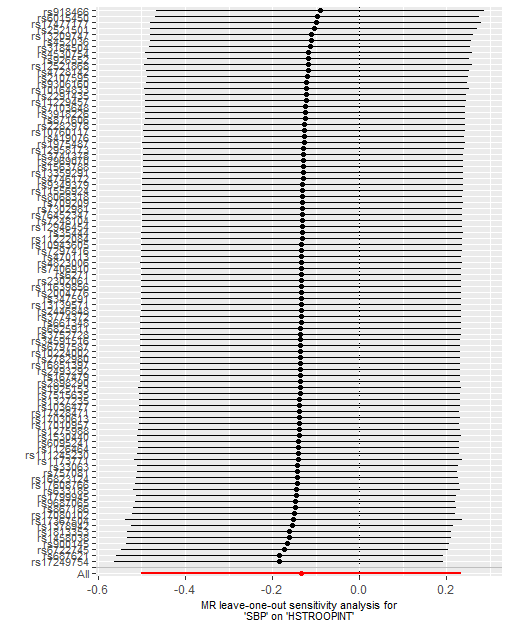 |
| --- | --- | --- |
| Leave-one-out plot for SBP effect on DSST | Leave-one-out plot for SBP effect on RAVLT | Leave-one-out plot for SBP effect on STROOP |
| 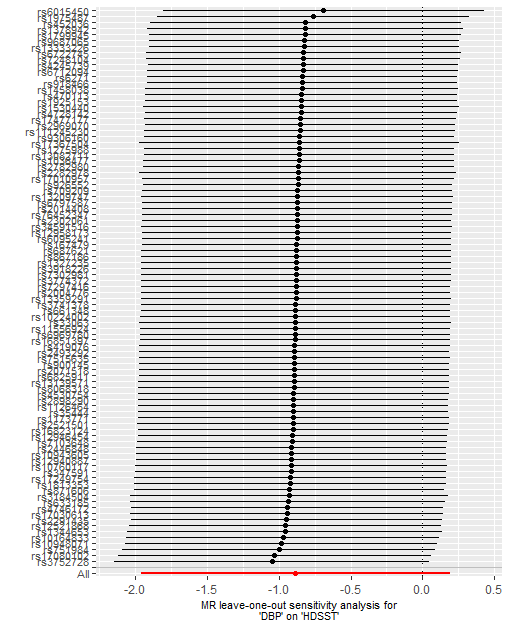 | 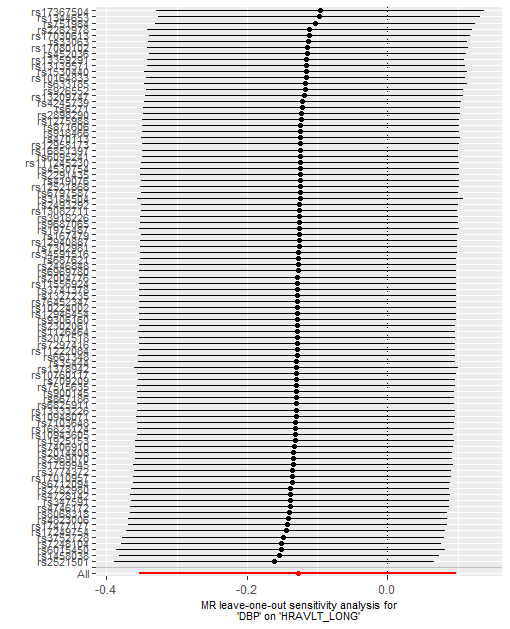 | 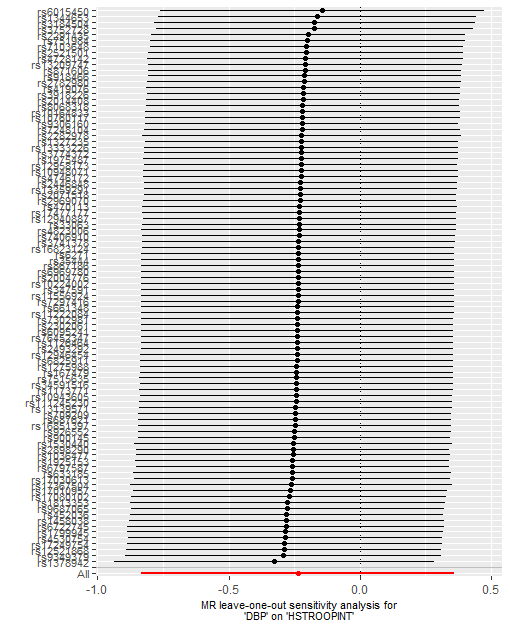 |
| Leave-one-out plot for DBP effect on DSST | Leave-one-out plot for DBP effect on RAVLT | Leave-one-out plot for DBP effect on STROOP |
| 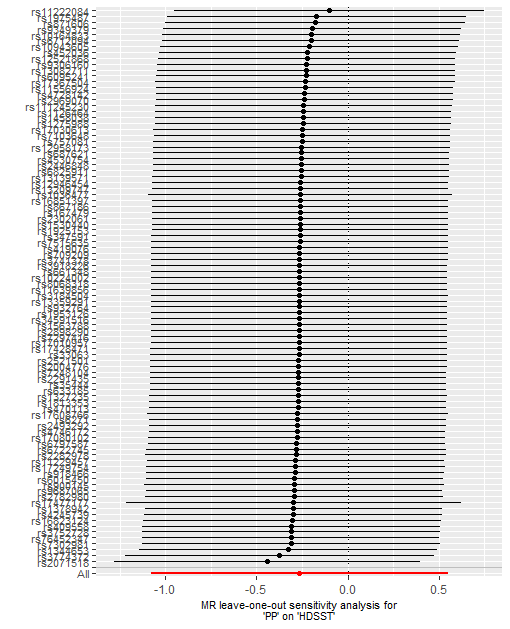 | 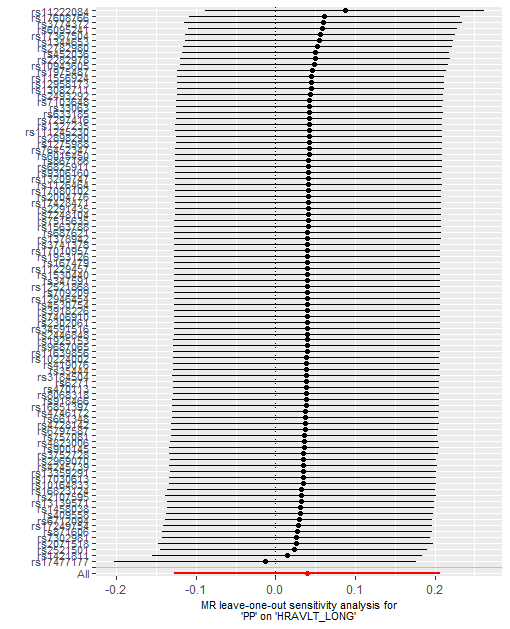 | 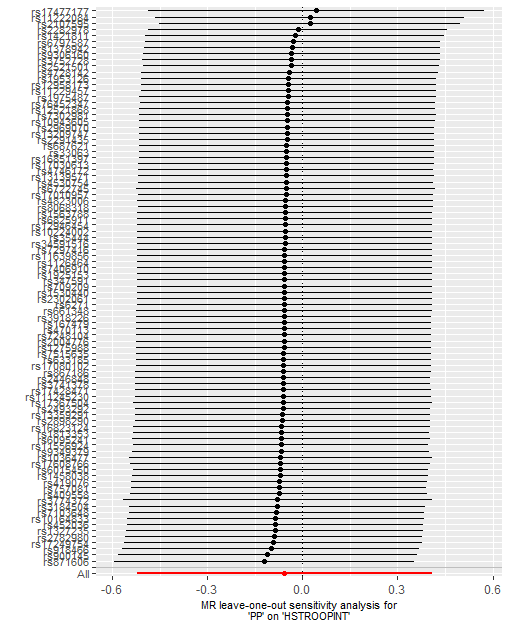 |
| Leave-one-out plot for PP effect on DSST | Leave-one-out plot for PP effect on RAVLT | Leave-one-out plot for PP effect on STROOP |
